# Metabolic and dietary features in kidney stone formers: nutritional approach

**DOI:** 10.1590/2175-8239-JBN-2020-0061

**Published:** 2020-06-01

**Authors:** Adamasco Cupisti, Claudia D’Alessandro

**Affiliations:** 1 University of Pisa, Department of Clinical and Experimental Medicine, Pisa, Italy

Kidney stone disease is not only a pathology of the urinary tract, but it is also closely associated to metabolic abnormalities and incorrect dietary habits. Rodrigues et al.^1^ reported the results of an observational study about the metabolic and dietary features of a Brazilian population of kidney stone patients. They found that kidney stone formers had a higher prevalence of obesity, diabetes, and hypertension together with higher serum levels of glucose and triglycerides than non-stone formers. These features are indicative of metabolic syndrome, which is an insulin resistance condition and it may be the link to nephrolithiasis. 

Insulin resistance increases the risk of kidney stone formation through changes in metabolism and transport of the kidney tubule cells. Actually, insulin resistance impairs the renal acidification process, namely ammonium production from L-glutamine in the proximal tubule cells, lowering urinary pH and citrate excretion^2^^,^ 3. These changes predispose the patient to uric acid and calcium oxalate supersaturation in urine, leading to both uric acid and calcium oxalate stone disease. In addition, kidney stone formers have a higher risk of diabetes, hypertension, and cardiovascular events, including myocardial infarction. This picture suggests that uric acid and calcium oxalate kidney stone disease should be viewed as a systemic disorder likely linked to metabolic syndrome^4^. Hence, dietary and lifestyle changes represent major strategies to be adopted for kidney stone patients in the clinical setting, which are not limited in preventing kidney stone recurrences but also metabolic syndrome and its related complications^5^.

Healthy dietary patterns, as the DASH or Mediterranean diet, represent a population-based substantial approach able to reduce the risk of cardiovascular events. The diets are rich in fruits, vegetables, whole grains, and low-fat dairy foods; lean meat, fish, poultry, nuts, and beans are abundant whereas red meat, added fats, processed foods, high-salt foods, and sugar-sweetened foods and beverages are limited. The healthy dietary pattern represents a lifestyle that also includes not smoking and physical activity. 

Both Mediterranean and DASH diets are rich in vegetables. A plant-based diet raises the concern of high intake of oxalate. Noori et al. performed a small study where participants were randomly assigned to receive a DASH or a low-oxalate diet. They found a trend towards an increase in urinary oxalate excretion but also a trend towards a reduction in calcium oxalate supersaturation in the DASH versus the low-oxalate diet group. This occurred because of an increased magnesium and citrate excretion and a higher urine pH in the DASH diet subjects^6^. 

A DASH diet may lower the risk of kidney stone formation by increasing urinary citrate excretion and urine volume. A weak association between higher DASH intake and lower urine relative supersaturation was found, so a possible role of unidentified stone inhibitors in dairy products and/or plants was suggested^7^.

Rodrigues et al. reported also that the diet of kidney stone formers showed a higher amount of protein and salt but lower calcium than in controls^1^. Probably, the higher percentage of diabetes in their group could have influenced patient’s food choices. In any case, stone formers usually do not have a good adherence to a DASH dietary pattern and it may predispose to kidney stone formation. In fact, high protein, high salt, and low calcium intakes are associated with increased risk of nephrolithiasis. When an abnormal intake is observed for one or more diet component, a nutrient-specific patient-based dietary intervention is warranted^5^.

High consumption of proteins, especially of animal origin, leads to increased urine excretion of calcium, oxalate and uric acid, and lower urine pH and citrate: all of these changes lead to a pro-lithogenic profile. Plant-origin proteins exert much less effects on these parameters: one more reason to prefer a plant-based diet. 

Dietary sodium load causes an increase in urinary calcium excretion. Thus, a restriction of dietary salt intake (i.e., a salt intake of 5-6 g/day as recommended in a healthy diet) should be suggested for primary and secondary prevention of calcium stone formation. 

A low calcium intake (400-500 mg/day) may decrease calciuria in calcium stone formers but it increases urine oversaturation of calcium-oxalate due to the increase of oxaluria coming from increased intestinal absorption. Therefore, a diet with a calcium content ≥1g/day is preferable, apart from a condition of diet-dependent hypercalciuria. Finally, a comprehensive dietary approach should include also interventions aiming to prevent or correct conditions that cause metabolic changes as overweight or obesity, dyslipidemia, diabetes, hypertension, etc^5^. 

In conclusion, the findings of Rodrigues et al. in Brazilian kidney stone formers confirm that metabolic syndrome and unhealthy dietary patterns are associated with kidney stone formation. This warns us that it is time to implement a more extensive metabolic assessment and correct nutritional imbalances in the clinical management of kidney stone patients. Promoting a healthy dietary pattern could be the first population-based intervention as a primary prevention strategy to reduce the risk of kidney stone formation and cardiovascular events. Patient-based nutrient-specific dietary manipulations can be added in a secondary prevention setting after the assessment of cardiovascular and kidney stone risk factors. 
